# Influence of fly ash filler on the mechanical properties and water absorption behaviour of epoxy polymer composites reinforced with pineapple leaf fibre for biomedical applications

**DOI:** 10.1039/d4ra00529e

**Published:** 2024-05-03

**Authors:** Santhosh Nagaraja, Praveena Bindiganavile Anand, Shivakumar H. D., Muhammad Imam Ammarullah

**Affiliations:** a Department of Mechanical Engineering, MVJ College of Engineering Bangalore 560067 Karnataka India; b Department of Mechanical Engineering, Nitte Meenakshi Institute of Technology Bangalore 560064 Karnataka India; c Department of Mechanical Engineering, Faculty of Engineering, Universitas Diponegoro Semarang 50275 Central Java Indonesia imamammarullah@gmail.com

## Abstract

This study explores the impact of fly ash (FA) filler on the mechanical, morphological, and water absorption properties of pineapple leaf fibre (PALF)-reinforced epoxy composites for biomedical applications. PALF, sourced from abundant agricultural waste, offers a sustainable alternative to synthetic fibres. Employing the hand layup process, varying wt% of FA (3%, 6%, and 9%) are incorporated into PALF-reinforced epoxy composites with different PALF concentrations (10%, 20%, and 30%). Mechanical assessments, including impact, flexural, and tensile strength, reveal that the introduction of up to 6 wt% FA enhances tensile strength by 65.3%, reaching its peak at this concentration. Flexural strength also improves by 31.9% with 6 wt% FA, while impact resistance reaches its maximum (74.18% improvement) at 9 wt% FA. Water absorption measurements demonstrate a decrease with increased FA content and exposure period, indicating enhanced water resistance. Scanning electron microscopy confirms the uniform distribution of FA, contributing to improved mechanical characteristics and water resistance. Optimality tests using Taguchi and response surface methodology (RSM) further confirm the experimental outcomes, emphasizing the potential of FA to enhance natural fibre-reinforced composites. This research suggests FA as a promising filler to elevate mechanical performance and water resistance in environmentally friendly composites.

## Abstract 2

This study explores the impact of fly ash (FA) filler on the mechanical, morphological, and water absorption properties of pineapple leaf fibre (PALF)-reinforced epoxy composites for biomedical applications.

## Graphical abstract

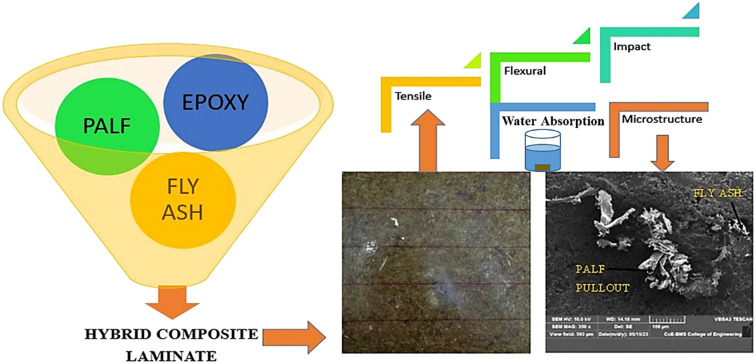

## 1. Introduction

There are several natural reinforcements that are extensively used for the synthesis of composites, but they have certain drawbacks in terms of water absorption characteristics.^1–5^ Thus, work has focused more on developing composites with filler materials that inhibit water absorption, while enhancing the strength required for several load-carrying structures: for example, the overhead stowage bins in airplanes, automotive dashboards *etc.*^6–9^ Several researchers have accomplished path-breaking research in the domain of natural composites, but the information available about the use of discernible filler materials is still in its incipient stage.^10–13^ Hence, the present work is a novel approach towards such an attempt. Natural fibres are recyclable, and offer sustainable and economical solutions as reinforcements in otherwise synthetic reinforcement dominated composites.^14–18^ The properties of composites fabricated using natural fibres as reinforcements are affected by fibre orientation, wt% of the fibre reinforcement and the bonding between the matrix and the fibres.^19–23^ The choice of suitable fibres for the fabrication of composites is also a major challenge, based on several attributes. In this regard, PALF is a discernible natural reinforcement, with excellent bonding strength that can be used as reinforcement. The use of PALF, owing to its renewable nature, cost-effectiveness, and advantageous mechanical qualities, enhances the strength of the composites.

Although natural reinforcements provide eco-compatibility and enhanced sustainability, their biodegradability has affected the characteristics of the composites. Hence, researchers across the globe have investigated various common methods for enhancing the characteristics of such composites.^24–27^ Two prevalent methods consist of fortifying the composites with organic or inorganic fillers to augment the durability of the matrix material, and hybridizing the composites with various types of fibre or filler to achieve a wide range of composite qualities.^28–31^ Hybridization involves combining with other natural or synthetic fillers to obtain a combination of their respective properties in the resulting composites, broadening their range of application. Various methods, such as layer-by-layer stacking, arranging different fibres in a single layer, varying the fibre orientation, and selective fibre placement, are employed for hybridization.^32–36^ At the nanoscale, nano-fillers can be added to create hybrid nanocomposites, with their properties influenced by the choice of nano-filler, manufacturing method, dispersion, and interaction between fibres and the matrix.^37–41^ The addition of FA, red mud and other discernible wastes causing threats to environment, as filler materials to a polymer matrix is a novel research area, since findings on the effectiveness of utilizing these discernible wastes from human activities are still in their nascent stage, and only incipient information is available. Typically considered inert materials, fillers improve the physical and mechanical properties of composites, leading to a better surface finish and mechanical performance.^42–46^ Inorganic fillers are preferred to achieve better characteristics. The impact of fillers on composite properties depends on their size, shape, aspect ratio, surface area, and dispersion within the composite.^47,48^

Fillers added to polymer matrices contribute to improved mechanical properties and reduced water absorption in fibre-reinforced composites due to their homogenous dispersion achieved by mechanical action.^49^ This ensures effective stress transfer between the matrix and fillers during loading, enhancing mechanical properties. However, an excessive amount of filler can lead to agglomeration and decreased bonding strength between the matrix and fibres, resulting in reduced mechanical strength.^50^ Effective utilization of fillers depends on their particle size, with smaller particles providing a higher surface area per unit weight and better interaction with the polymer matrix. Fillers offer high aspect ratios and specific surface areas, making them ideal for enhancing various properties of composites.^51^

Understanding how the addition of fly ash affects the mechanical properties and water absorption behavior of polymer composites can aid in developing materials suitable for biomedical devices, implants, or prosthetics. It provides insights into the durability and stability of these materials in biological environments. Composites developed by reinforcing fillers through advanced manufacturing methods exhibit high-performance characteristics. Integrating nanotechnology principles into composite development can significantly enhance overall performance. Many countries have already started utilizing natural filler-based composites in various applications, such as rail and decking products, car components, architectural moldings, and more.^52–54^ Inorganic fillers include various materials like zinc oxide, alumina, calcium carbonate, and silica, while organic fillers consist of particulates derived from plants and animals. The quantity of filler added to the polymer matrix depends on the type of filler and matrix used, usually varying between 4% and 5% by weight.^55^ Overall, fillers offer great potential for improving the mechanical performance of composites and advancing innovative materials for diverse applications. Continued research in this area is crucial for the development of sustainable and high-performance composite materials. In this regard, the objectives of the present work are framed with a clear-cut focus on the development of sustainable composites making use of PALF reinforcements and FA filler material. The use of FA for the development of sustainable polymer composites provides a suitable method for utilizing the discernible by-product of the combustion of coal in thermal power plants, which is a major threat to the environment across the globe. The dumping of FA in landfill is causing serious concerns with respect to soil, air and water pollution. Thus, there is a need to develop eco-compatible methods of utilizing FA to develop composites that have better characteristics and are sustainable.

## 2. Materials and methods

**Fig. 1:**
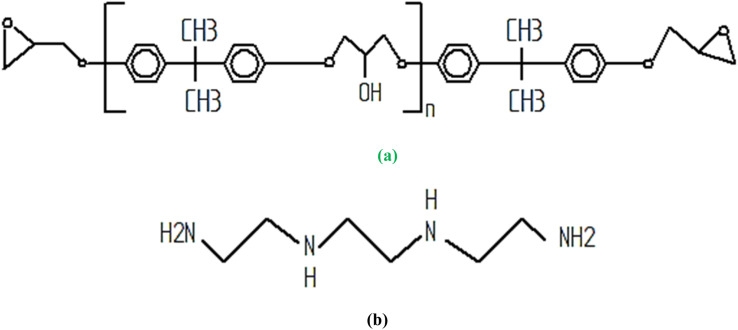
Specific molecular structure of (a) LY-556 epoxy and (b) HY-951 hardener.

In this study, LY-556 epoxy/HY-951 hardener, acquired from SS Impex, Bangalore, was chosen as the matrix material. Fig. 1 gives the specific molecular structure of the epoxy and hardener used in the present work. The specific molecular structures of LY-556 epoxy resin and HY-951 hardener are proprietary and typically not publicly disclosed by the manufacturers. However, epoxy resins like LY-556 usually comprise linear polymers containing epoxide functional groups (–CH_2_–CH_2_–O–), offering versatile properties tailored to diverse applications. Conversely, hardeners such as HY-951 often contain amine functional groups (–NH_2_) that facilitate crosslinking with epoxy resins, leading to a robust three-dimensional network structure during curing. Epoxy resins are prized for their adhesive strength, chemical resistance, and mechanical integrity, while hardeners play a crucial role in enhancing the overall performance of the material. Though the density and other characteristics of these materials can vary widely depending on their formulation and intended use, epoxy resins typically exhibit densities ranging from 1.1 to 1.4 g cm^−3^, while hardeners may fall within the range of 0.8 to 1.2 g cm^−3^.

Pineapple leaf fibre (PALF) chopped to fine lengths in the range of 8 mm to 12 mm with diameters in the range of 10 μm to 25 μm obtained from ‘Greentech Fibres’, Chennai, was used as reinforcement. The cellulose content of PALF typically ranges from 65% to 75% of its total composition. The lignin content, on the other hand, generally constitutes about 20% to 25% of PALF's composition. However, PALF fibres require suitable pretreatment with silane-based coupling agents. In FA–PALF–epoxy composites, coupling agents are pivotal to augment the mechanical properties through facilitating enhanced adhesion among the composite constituents. Given the disparate surface chemistries of FA, PALF, and the epoxy matrix, effective bonding necessitates intermediary compounds. Coupling agents, like silane-based varieties, serve this purpose by forming chemical bonds with the organic and inorganic components. These agents feature functional groups capable of reacting with hydroxyl groups on FA and PALF surfaces, as well as with epoxy resin molecules, thereby fostering improved compatibility. This chemical bridging enhances stress transfer across interfaces, leading to heightened mechanical properties, such as tensile and flexural strength, along with increased impact resistance.

Fly ash of particulate sizes in the range of 40 μm to 75 μm sourced from the KPCL plant, Raichur, Karnataka, was further used as filler material to enhance the performance of the PALF-reinforced composites. FA, rich in silicon dioxide, alumina, and iron oxide, was identified for its potential to improve mechanical and morphological properties while addressing environmental concerns related to its disposal. Through initial trials and a literature review, the weight percentages of PALF (ranging from 10% to 30%) and FA (ranging from 3% to 9%) were determined. It was found that PALF below 10 wt% led to reduced strength characteristics, while exceeding 30 wt% caused coring and agglomeration, resulting in the formation of voids. Similarly, FA content below 3 wt% had minimal impact on strength, but when it exceeded 9 wt% it led to debonding of fibres due to FA agglomerates in the matrix. The experimental design followed an L9 ‘orthogonal array’, with optimal weight percentages determined to be 20 wt% PALF and 6 wt% FA. Table 1 gives the wt% of the constituents for different composite specimens.

**Table 1:** Wt% of constituents for different specimen designations

Specimen designation	Wt% of PALF	Wt% of FA	Wt% of LY-556 epoxy	Wt% of HY-951 hardener
P10F3	10	3	78.3	8.7
P20F3	20	3	69.3	7.7
P30F3	30	3	60.3	6.7
P10F6	10	6	75.6	8.4
P20F6	20	6	66.6	7.4
P30F6	30	6	57.6	6.4
P10F9	10	9	72.9	8.1
P20F9	20	9	63.9	7.1
P30F9	30	9	54.9	6.1
Neat epoxy	0	0	90	10
Neat epoxy + F6	0	6	84.6	9.4
Neat epoxy + P20	20	0	72	8

**Fig. 2:**
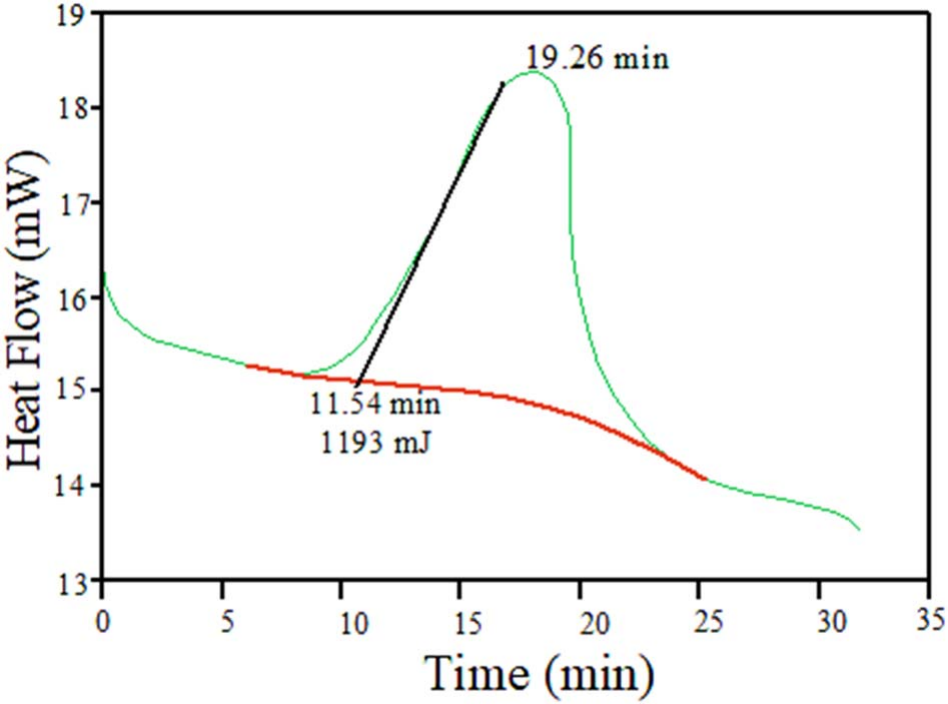
Differential scanning calorimetry (DSC) of epoxy curing.

**Fig. 3:**
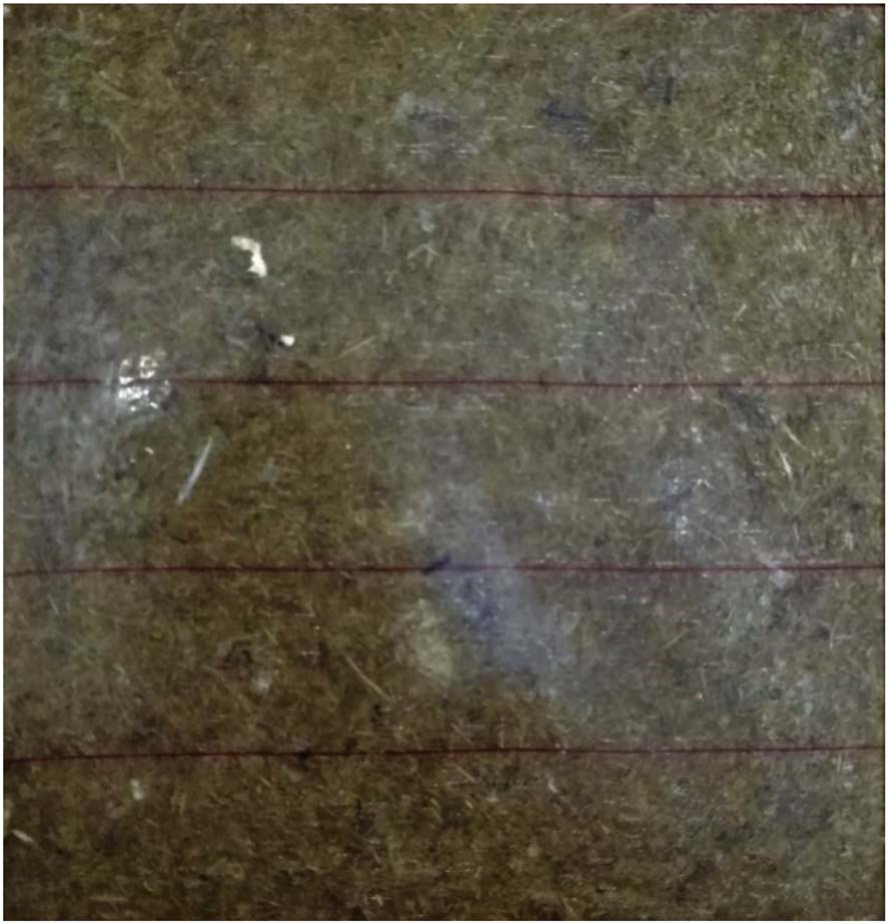
Photograph of the composite laminate fabricated in the present work.

The composites are fabricated using ultrasonic-assisted stirring and hand layup techniques under room-temperature conditions on a dry granite slab. This process entails manually mixing chopped pineapple fibres and FA with the epoxy hardener matrix through stirring. Prior to pouring the mixture into the mold, the mold surface is cleaned thoroughly and a release agent is applied to prevent sticking. Each layer is meticulously wetted with epoxy resin using brushes and rollers to ensure proper adhesion and to eliminate air pockets or voids. Layer-by-layer application continues until the desired thickness and laminate structure are achieved. After consolidating the layers and removing trapped air bubbles, the composite is allowed to cure according to the recommended time and temperature (24 h and 90 °C) provided by the epoxy resin manufacturer. Once fully cured, the composite part is demolded, trimmed, and finished to achieve the desired final appearance and smoothness, following the safety precautions and guidelines outlined by the resin and hardener manufacturers. Fig. 2 depicts the DSC of epoxy curing while Fig. 3 shows a photograph of the composite laminate fabricated in this study. The DSC curve exhibits a distinct curing reaction peak occurring at 19.26 minutes. This peak indicates the onset of epoxy resin curing, marking the initiation of the crosslinking reaction between the epoxy resin and the curing agent. The corresponding heat flow observed at this peak is measured at 1193 mJ, signifying the amount of heat flow during the curing process.

The composite laminate undergoes cutting using abrasive jet machining to meet ASTM standard sizes for tensile, flexural, and impact tests. Tensile tests adhere to ASTM D3039/D3039M standards, with specimens prepared with dimensions of 50.8 mm gauge length, 12.7 mm width, and 3.2 mm thickness, tested on Instron 3360 series equipment. Flexural tests follow ASTM D790/D790 M standards using 127 mm long, 12.7 mm wide, and 3.2 mm thick specimens, while Izod impact testing conforms to ASTM D256 standards with specimens of 63.5 mm length, 12.7 mm breadth, and 3.2 mm thickness. The results are recorded and analyzed to assess the impact of FA on characteristics. Water absorption behavior is investigated according to ASTM D 570-98 standards, with rectangular samples of 75 mm length, 25 mm width, and 3.2 mm thickness subjected to pre-treatment drying followed by immersion in distilled water. Weight measurements at intervals, alongside calculations using eqn (1), determine water absorption%.

1 (*W*_*t*_ − *W*_0_)/*W*_0_ × 100

where *W*_*t*_ is the weight of the sample at time *t*, and *W*_0_ is the initial weight recorded before immersion in water.

The procedure is repeated for all the composite specimens to determine the water absorption for different wt% of fibre and filler contents and for different durations of time.

## 3. Results and discussion

The results presented in Table 2 give the mechanical properties of pineapple leaf fibre (PALF) and FA reinforced epoxy composites with varying wt% of FA (ranging from 3 wt% to 9 wt%) and pineapple leaf fibre (ranging from 10 wt% to 30 wt%). Additionally two unique combinations of neat epoxy + F6 and neat epoxy + P20 are characterized to understand the impact of unitary inclusions of FA and PALF, respectively, on the characteristics of the composites.

**Table 2:** Mechanical properties of pineapple leaf fibre and FA reinforced epoxy composites

Specimen designation	UTS (MPa)	Young's modulus (GPa)	Flexural strength (MPa)	Flexural modulus (GPa)	Impact strength (kJ m^−2^)
P10F3	56.4	1.8	121.9	3.6	23.49
P20F3	61.2	2.1	131.5	3.9	24.56
P30F3	58.9	1.9	126.8	3.8	25.93
P10F6	73.2	2.9	154.7	4.3	29.21
P20F6	86.6	3.6	165.3	4.6	33.56
P30F6	78.4	3.1	163.6	4.5	35.39
P10F9	65.7	2.5	133.8	3.9	37.83
P20F9	69.4	2.8	143.9	4.1	39.68
P30F9	73.5	2.9	149.2	4.2	34.13
Neat epoxy	52.4	1.5	125.3	3.7	22.78
Neat epoxy + F6	70.3	2.8	142.6	4	32.81
Neat epoxy + P20	65.8	2.5	132.7	3.9	24.64

### 3.1 Ultimate tensile strength (UTS)

**Fig. 4:**
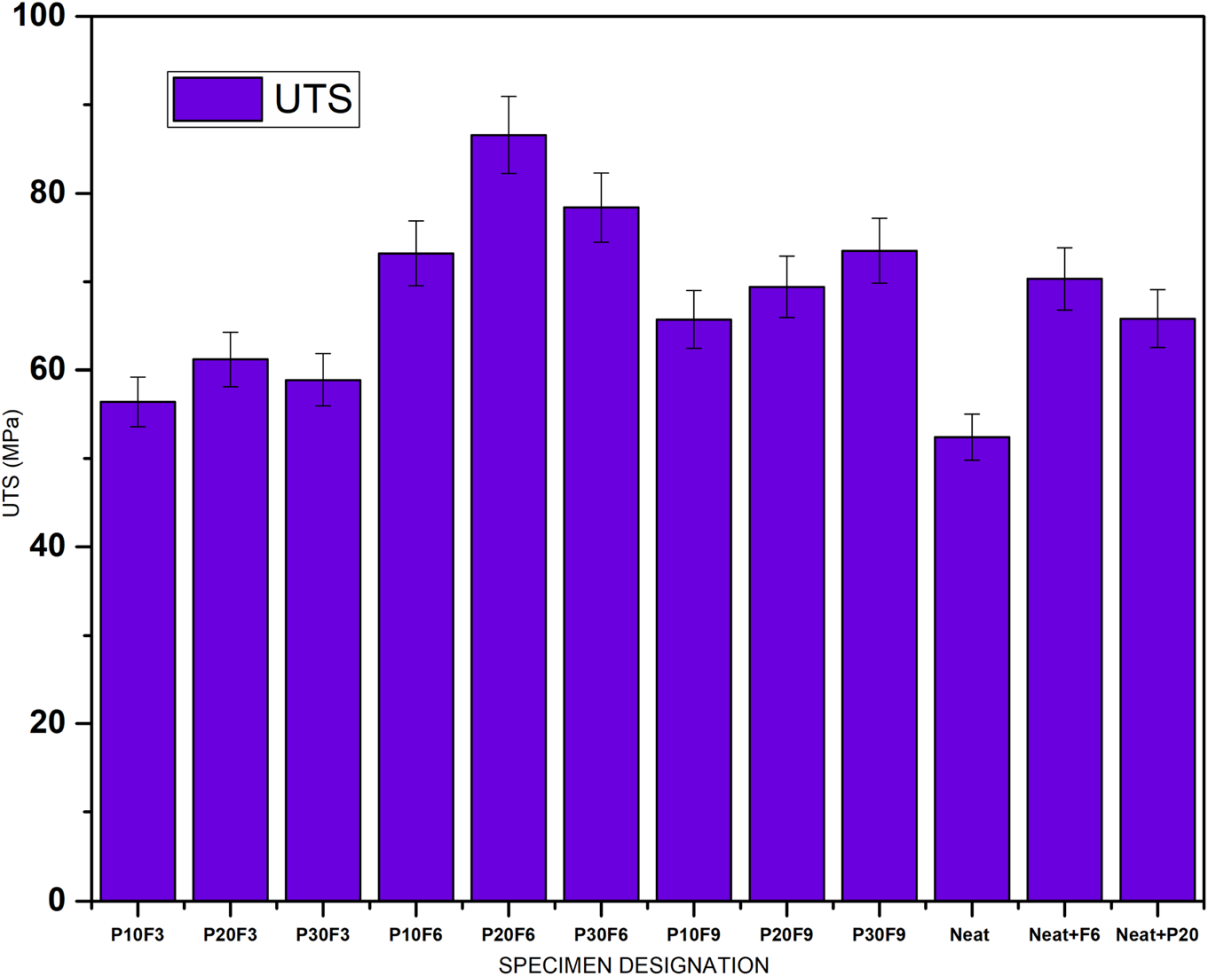
Ultimate tensile strength (UTS) for different specimens.

As the wt% of pineapple leaf fibre increases beyond 20 wt%, there is a general trend of decrease in the UTS of the composites. This is likely due to the intrinsic nature of natural fibres, which have lower tensile strength compared to synthetic fibres or other reinforcing materials. On the other hand, the addition of FA seems to improve the UTS. Overall, the composites with higher FA content and lower pineapple leaf fibre content tend to exhibit higher UTS. P20F6 exhibited the highest tensile strength, showcasing an improvement of 65.3% compared to neat epoxy. Fig. 4 gives a comparative bar chart for UTS for different wt% of PALF and FA.

### 3.2 Young's modulus

**Fig. 5:**
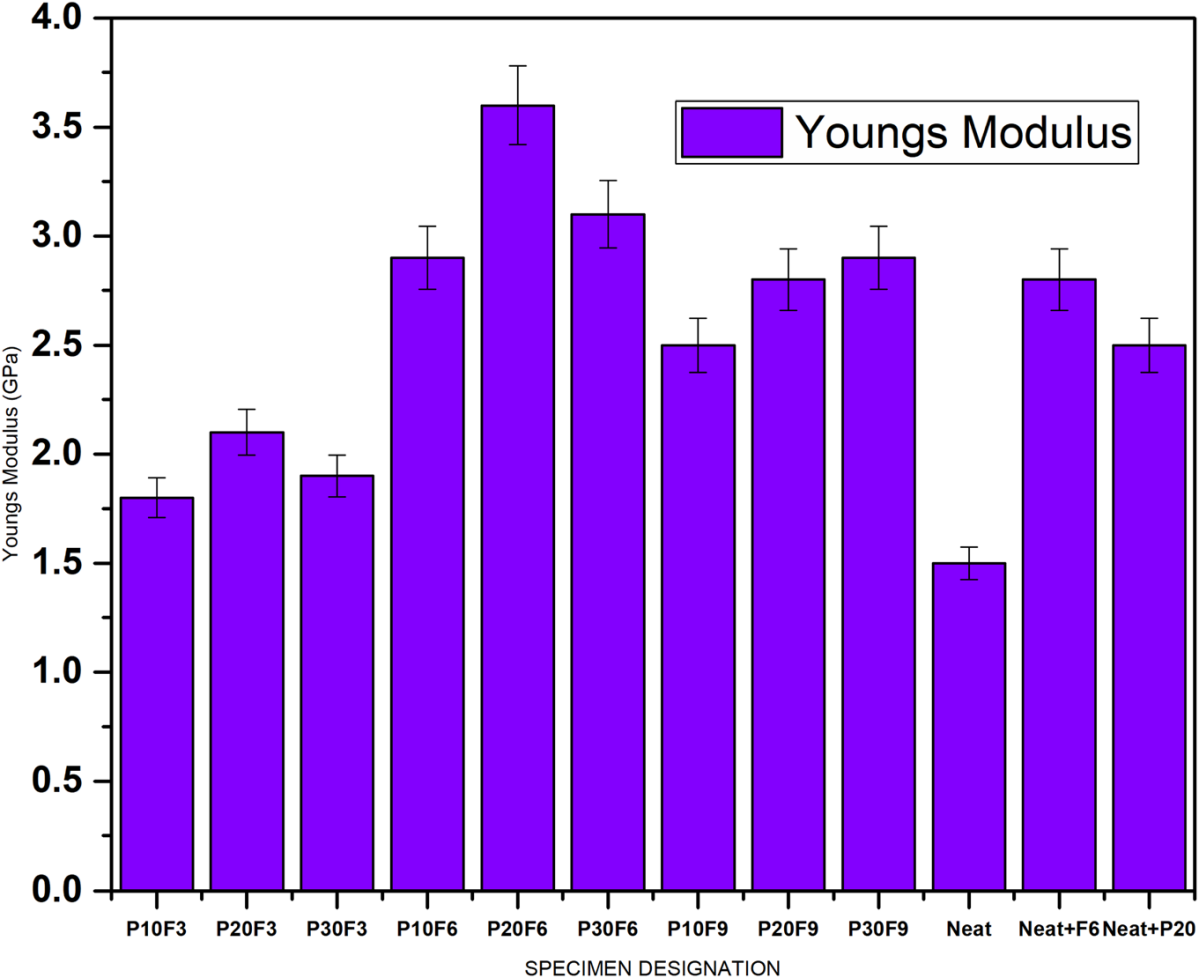
Young's modulus for different specimens.

The results show that increasing the FA beyond 6 wt% results in a decrease in Young's modulus. This can be attributed to the fact that natural fibres are generally less stiff than synthetic fibres. FA, on the other hand, is observed to have a favourable impact on Young's modulus, suggesting that it functions as a reinforcing filler to increase the stiffness of the composite. Composite P20F6 has the greatest stiffness, measuring 3.6 GPa for Young's modulus. Fig. 5 gives a comparative bar chart for Young's modulus for different wt% of PALF and FA.

### 3.3 Flexural strength and flexural modulus

**Fig. 6:**
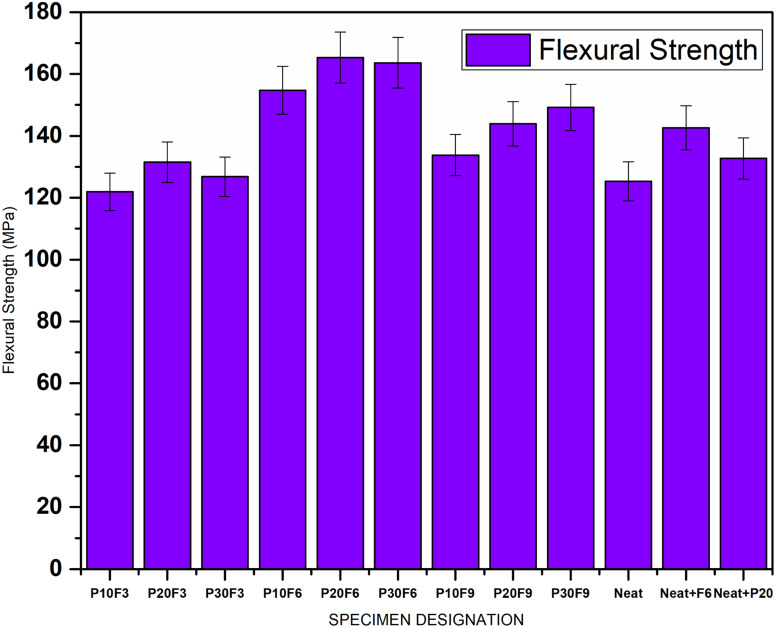
Flexural strength for different specimens.

**Fig. 7:**
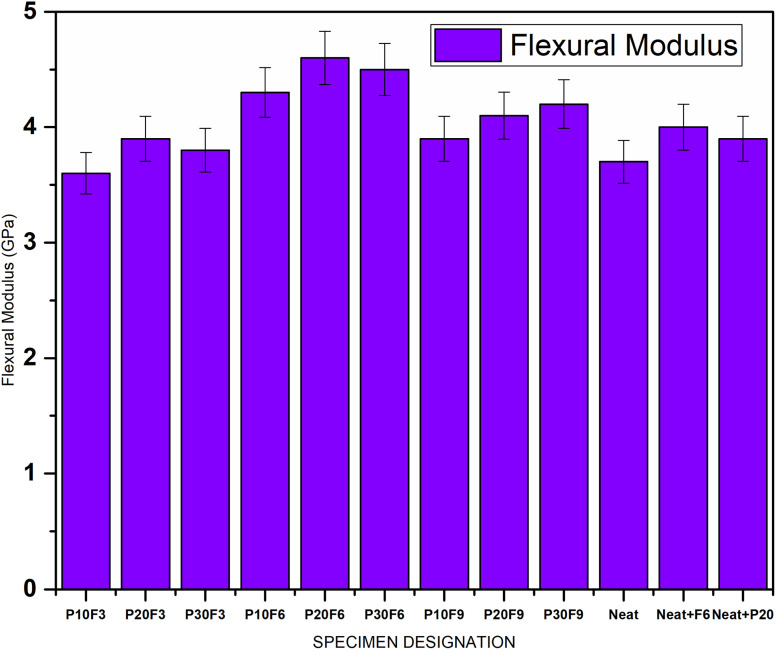
Flexural modulus for different specimens.

Flexural strength and flexural modulus are important properties in determining the ability of a composite to withstand bending or flexing loads. The results indicate that increasing the wt% of pineapple leaf fibre leads to a slight decrease in both flexural strength and flexural modulus. This is consistent with the trend observed in the UTS, where the lower strength of natural fibres affects these properties. On the other hand, as seen in the UTS and Young's modulus, the addition of FA seems to have a positive effect on flexural strength and flexural modulus, contributing to the enhanced resistance of the composite to bending. P20F6 displayed a maximum flexural strength of 165.3 MPa, demonstrating a 31.9% enhancement compared to neat epoxy. Fig. 6 and 7 give comparative bar charts for flexural strength and flexural modulus for different wt% of PALF and FA, respectively.

### 3.4 Impact strength

**Fig. 8:**
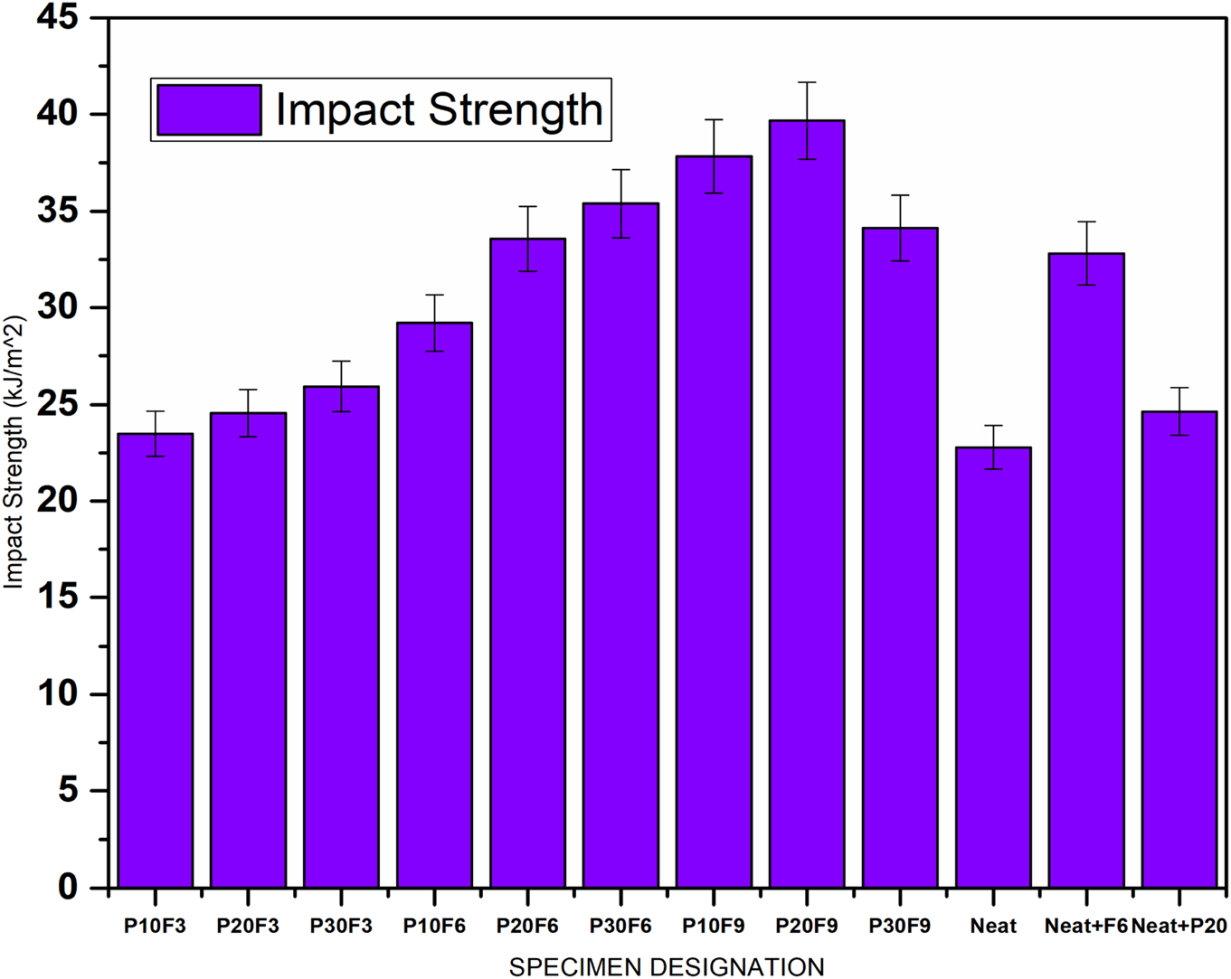
Impact strength for different specimens.

Impact strength measures the ability of a material to absorb energy during impact loading. Interestingly, the impact strength does not follow a consistent pattern with varying wt% of pineapple leaf fibre and FA. For instance, the impact strength increases with the addition of pineapple leaf fibre and FA content in the P20F3, P30F3, P10F6, P20F6, P30F6, P10F9 composites, and is a maximum for the P20F9 composite with a value of 39.68 kJ m^−2^ exhibiting an improvement of 74.18% compared to neat epoxy, but it decreases slightly in the P30F9 composite. Such behavior could be attributed to the complex interplay of factors like fibre dispersion, interfacial adhesion, and the orientation of fibres in the composite. The impact strength of composite materials is intricately linked to the uniform distribution of reinforcing fibres within the matrix (fibre dispersion), the strength of the bond between fibres and the matrix material (interfacial adhesion), and the orientation of these fibres. Enhanced impact resistance results from a more uniform fibre dispersion that mitigates stress concentration, robust interfacial adhesion promoting efficient stress transfer, and a strategic fibre orientation that bolsters the ability of the material to withstand impact loads. Notably, these factors are not independent but interact synergistically, and a holistic approach to optimizing these parameters can yield substantial improvements in impact resistance, rendering composite materials highly versatile for a wide array of applications.^56,57^Fig. 8 gives a comparative bar chart for impact strength for different wt% of PALF and FA.

Research conducted by P B Anand *et al.*^20^ most likely included an examination of how various composite materials responded when subjected to controlled impact forces. These impact tests would have entailed applying specific levels of force to the materials and then assessing how well they resisted breaking or undergoing deformation as a result of these impacts. The outcomes of these tests are in line with the findings of the present work, wherein the fibre bonding facilitated by the pretreatment and addition of filler material endures the impact forces imposed upon it. In essence, the study aimed to evaluate the ability of the composite materials to withstand and absorb the energy from applied impact loads, and this information was likely presented through numerical data, graphs, or tables that showcased the specific impact strength values. Such results are crucial in determining the suitability of these materials for various applications where impact resistance is a significant factor.

### 3.5 Test for optimality

**Fig. 9:**
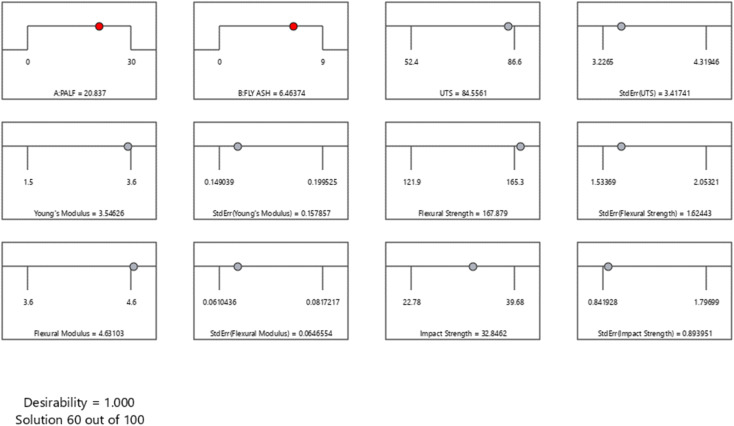
Results of the 60th optimality trial run and the standard error.

The provided data is associated with a design experiment, where various factors and responses were measured across different trials. From the experimental trials, it is evident that the 29th trial seems to be the optimized trial for the tested factors and the objectives of the experiment. In the present work, Design-Expert is used for designing experiments and optimizing processes by analyzing complex experimental data. To interpret the 60th optimality run number, with 20.837 wt% PALF, and 6.46374 wt% FA, we observed optimized response variables. The test for optimality checks for desirability values for indications of goal fulfillment, assessment of predefined optimization criteria, and consideration of standard errors for measurement precision. The specific details and goals of the experiment have a significant impact on interpretation of the results, and the features are utilized to delve deeper into the results and potentially identify optimal conditions based on defined criteria. Table 3 gives the outcomes of the 60th optimality run that is close to the 20 wt% PALF and 6 wt% FA, which gives relatively better mechanical characteristics, while Fig. 9 gives the results of the optimal trial run and the standard error.

**Table 3:** Outcomes of the 60th optimality trial run

Optimality run	PALF	FA	UTS	Young's modulus	Flexural strength	Flexural modulus	Impact strength	Desirability
60	20.837	6.464	84.556	3.546	167.879	4.631	32.846	1

**Fig. 10:**
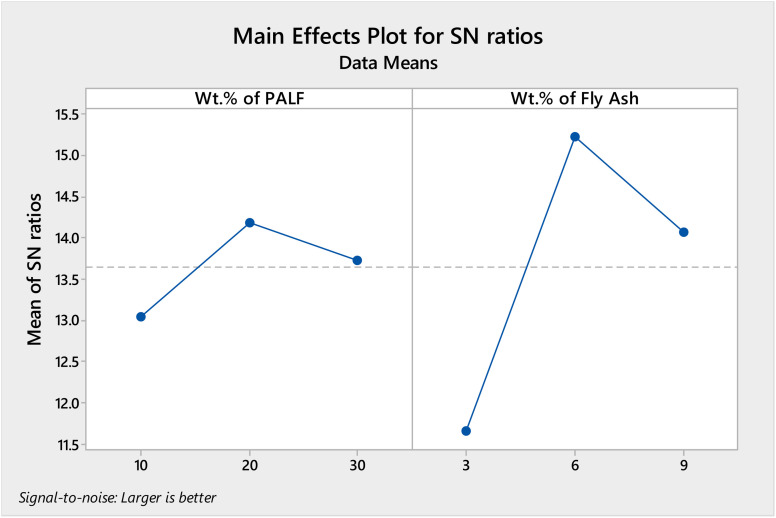
Main effects plot for SN ratios for UTS (MPa).

**Fig. 11:**
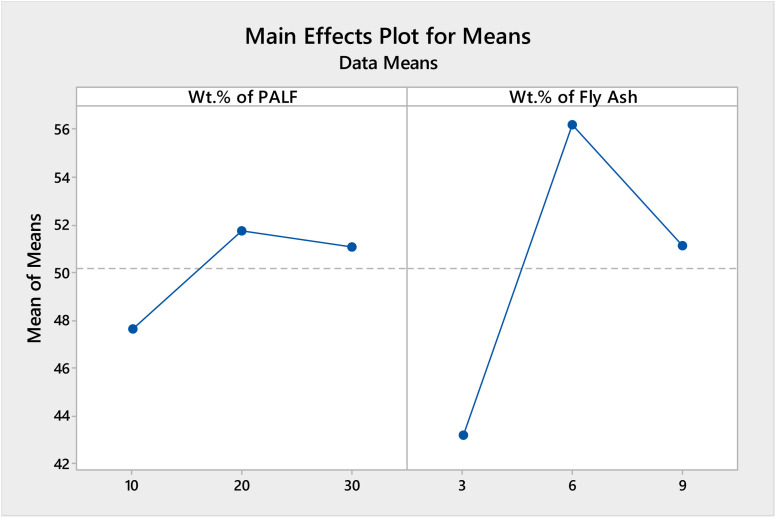
Main effects plot for means for UTS (MPa).

The pilot set of Taguchi analysis and response surface methodology (RSM) was accomplished for the tensile strength to ascertain the outcomes of the optimality test, *i.e.*, to analyze the impact of wt% of PALF and FA on the characteristics of the composites. The response tables for signal to noise (SN) ratios and means are given in Tables 4 and 5, respectively, and plots of the main effects are represented in Fig. 10 and 11, respectively.

**Table 4:** Response table for SN ratios for UTS (MPa)

Level	Wt% of PALF	Wt% of FA
1	13.04	11.66
2	14.18	15.23
3	13.73	14.07
Delta	1.15	3.57
Rank	2	1

**Table 5:** Response table for means for UTS (MPa)

Level	Wt% of PALF	Wt% of FA
1	47.68	43.19
2	51.79	56.2
3	51.08	51.17
Delta	4.1	13.01
Rank	2	1

From the response tables and main effects plot, it is evident that level 2 gives the maximum UTS in MPa for the “larger is better” ratio for SN ratios and the main effects plot with the wt% of FA being ranked 1, as the major contributor to the UTS, followed by wt% of PALF.

**Fig. 12:**
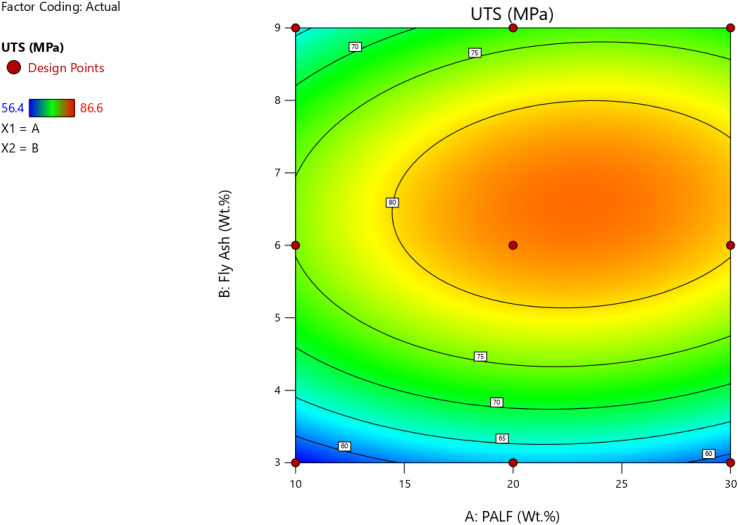
Surface plot for UTS (MPa) for varying wt% of FA and PALF.

**Fig. 13:**
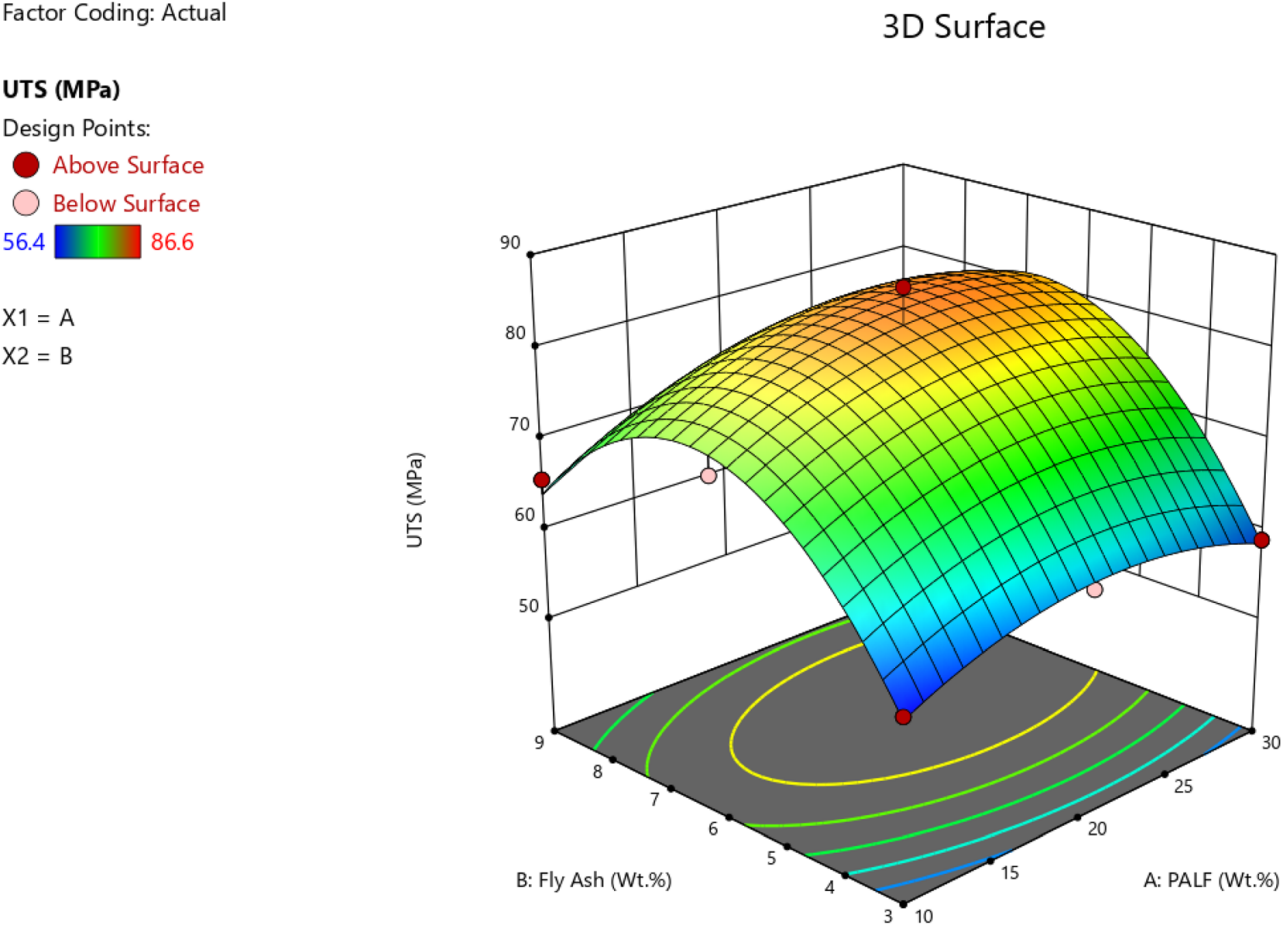
3D Contour plot for UTS (MPa) for varying wt% of FA and PALF.

**Fig. 14:**
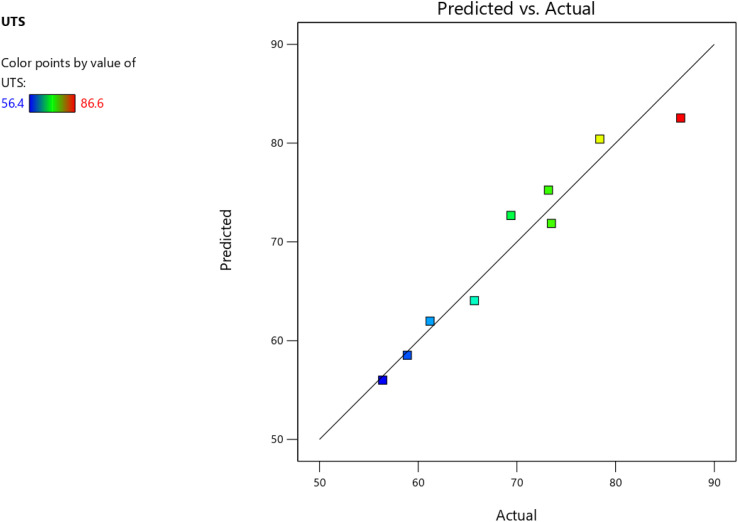
Predicted *vs.* actual for UTS (MPa) for varying wt% of FA and PALF.

Further, the RSM was accomplished in Design-Expert software to ascertain the optimality with reference to the wt% of PALF and FA for UTS in MPa for the composites. Fig. 12 and 13 give the surface plot and 3D contour plot for the different design points for varying wt% of PALF and FA. From the plots, it is evident that the maximum UTS represented by red-coloured zones is observed for the range of 6 wt% to 6.5 wt% for FA filler, and 20 wt% to 22 wt% for PALF reinforcements. The predicted *vs.* actual plots for the UTS (MPa) are in close correlation with each other, as shown in Fig. 14.

Table 6 gives the ANOVA table, where the *F*-value of the model of 10.45 clearly shows that the model is significant. There is only a 4.09% chance that an *F*-value this large could occur due to noise. Further, *P*-values <0.05 indicate that the model terms are significant. In this case *B* and *B*^2^ are significant model terms. *P*-values >0.1 indicate that the model terms are not significant. If there are many insignificant model terms (not counting those required to support the hierarchy), model reduction may improve your model.

**Table 6:** ANOVA table for UTS (MPa) from RSM

Source	Sum of squares	d*f*	Mean square	*F*-Value	*p*-Value	Model prediction
Model	726.39	5	145.28	10.45	0.0409	Significant
A-PALF	40.04	1	40.04	2.88	0.1882	
B-FA	171.74	1	171.74	12.36	0.0391	
AB	7.02	1	7.02	0.5053	0.5285	
*A* ^2^	44.49	1	44.49	3.20	0.1715	
*B* ^2^	463.09	1	463.09	33.32	0.0103	
Residual	41.70	3	13.90			
Cor total	768.08	8				

Table 7 gives the fit statistics, where the predicted *R*^2^ of 0.5672 is not as close to the adjusted *R*^2^ of 0.8552 as one might normally expect; *i.e.* the difference is more than 0.2. This may indicate a large block effect or a possible problem with the model. The critical factors for fit statistics to consider are model reduction, response transformation, outliers, *etc.* All empirical models are tested by doing confirmation runs. Adeq precision measures the signal to noise ratio. A ratio greater than 4 is desirable. The ratio of 8.719 indicates an adequate signal. This model can be used to navigate the design space.

**Table 7:** Fit statistics for UTS (MPa) from RSM

Std. dev.	3.73	*R* ^2^	0.9457
Mean	69.26	Adjusted *R*^2^	0.8552
C.V.%	5.38	Predicted *R*^2^	0.5672
		Adeq precision	8.7195

The regression equation obtained from the fit statistics for UTS (MPa) obtained from RSM is given by eqn (2).

2 UTS = −7.56 + 1.88 × *A* + 21.19 × *B* + 0.044 × *A* × *B* − 0.047 × *A* × *A*−1.69 × *B* × *B*

where *A* is coded for PALF in wt%, while *B* is coded for fly ash in wt%.

A confirmation test was accomplished to ascertain the authenticity of the model and its predictions with the outcomes. Table 8 gives the results of the confirmation test.

**Table 8:** Results of confirmation test

PALF (wt%) *A*	FA (wt%) *B*	Experimental UTS (MPa)	Predicted UTS (MPa)	% error
21	6.5	85.1	83.5315	1.84

From the table, it is evident that the predicted UTS, up to the fourth decimal is in close agreement with the experimental UTS, and the RSM model evolved using Design-Expert is thus validated with the optimality test results and the Taguchi model evolved using Minitab Software.

### 3.6 Comparison with neat epoxy, neat epoxy + F6 and neat epoxy + P20

The investigation explored epoxy composites reinforced with PALF and FA as an eco-friendly alternative to conventional synthetic composites, noting the inherent strength and stiffness of PALF and the potential of FA as a composite filler. The study compared the mechanical properties of various epoxy composites with differing compositions of FA and PALF, including neat epoxy variants for reference. The results indicated that the P20F6 composite exhibited the highest overall performance, displaying superior flexural strength (165.3 MPa), flexural modulus (4.6 GPa), and impact strength (33.56 kJ m^−2^). Although the P10F6 and P30F6 composites also performed well, P20F6 showed slightly better results. All the composites showed enhanced mechanical properties compared to neat epoxy, underscoring the effectiveness of PALF and FA as reinforcing materials. The choice of optimal reinforcement–filler combinations depends on specific application requirements and desired trade-offs between strength, stiffness, and impact resistance. Further optimization and characterization may be necessary for the precise adjustment of composite characteristics for specific technical applications. The optimal wt% of fibre and filler, *viz.*, the P20F6 composite, demonstrates the best overall performance, making it suitable for applications prioritizing these properties. Further, the optimality test concluded that 20.837 (approx. 21) wt% PALF and 6.46374 (approx. 6.5) wt% FA closely predicted outcomes in close correlation with the experimental findings, further reducing the value gradient to obtain the optimal proportion of reinforcement and filler for the composite.

### 3.7 Water absorption

The water absorption readings were closely monitored for an interval of 24 hours for a duration of 5 days, and the data is recorded and tabulated in Table 9. A duration of 5 days was considered as the initial set of pilot experiments, revealing that water absorption tends to become almost constant beyond 5 days.

**Table 9:** Water absorption readings for every 24 hours interval for a duration of 5 days

Specimen designation	% water absorption				
	Day 1	Day 2	Day 3	Day 4	Day 5
P10F3	5.4	4.9	4.7	4.5	3.4
P20F3	6.2	5.8	5.5	4.9	4.3
P30F3	8.9	7.8	6.8	5.2	4.1
P10F6	2.3	1.6	1.5	1.3	1.1
P20F6	4.6	3.9	3.4	3.2	3.2
P30F6	6.4	7.1	6.6	5.6	4.3
P10F9	3.2	2.9	2.7	2.3	2.2
P20F9	5.4	4.5	3.9	3.1	2.8
P30F9	7.5	5.9	5.2	4.1	3.5
Neat epoxy	2.4	1.5	1.3	1.2	1.1
Neat epoxy + F6	3.3	2.8	2.6	2.5	2.4
Neat epoxy + P20	4.3	3.9	3.8	3.6	3.4

**Fig. 15:**
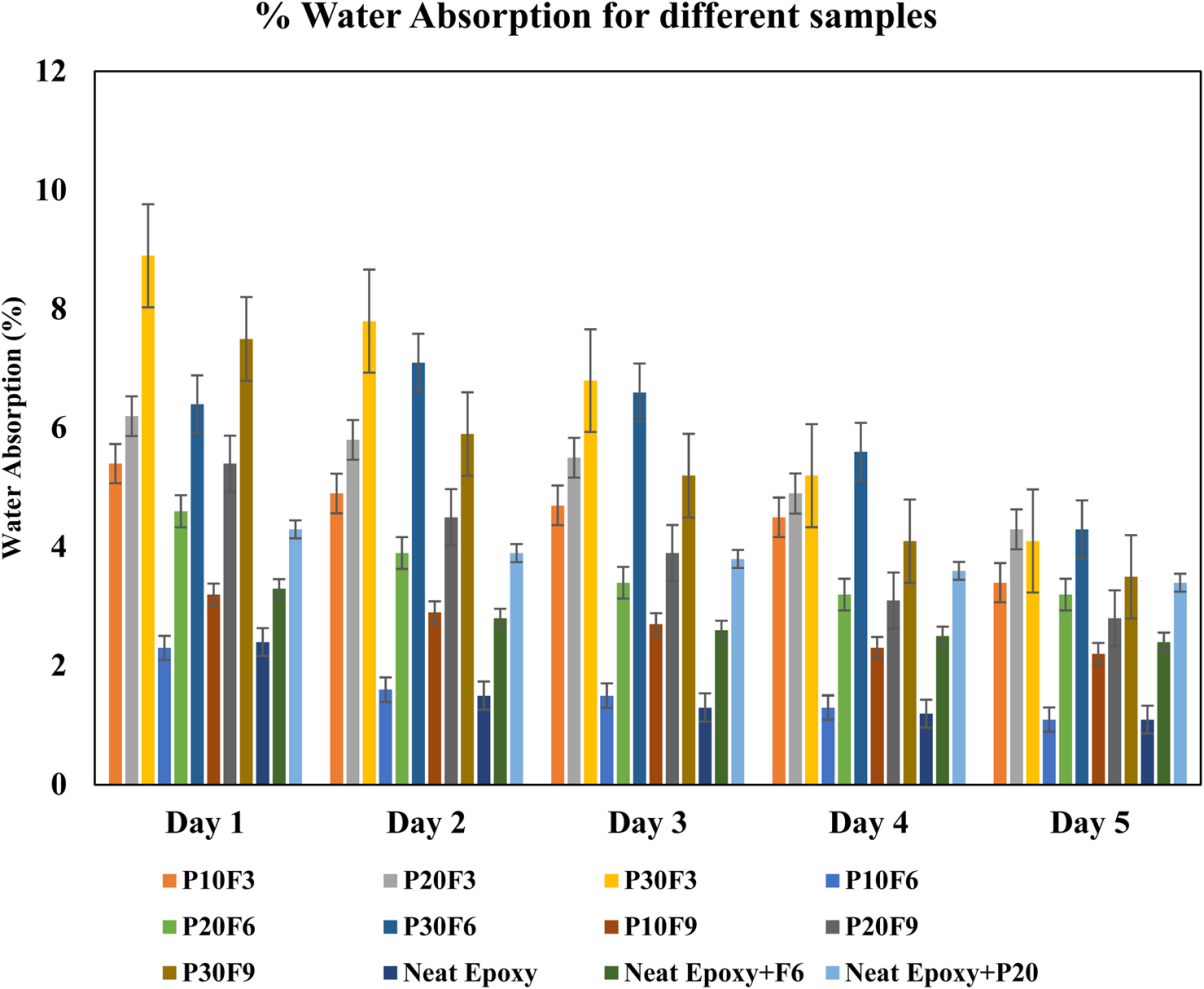
% water absorption for different samples for every 24 h interval over a period of 5 days.

The provided data outlines water absorption percentages for various specimens. Across specimens such as P10F3, P20F3, and P30F3, an increase in the wt% of FA corresponds to a general decrease in water absorption, indicative of the filler effect that diminishes overall material porosity. This trend persists in specimens like P10F6, P20F6, and P30F6, where a higher FA content results in a decline in water absorption, attributed to the filling effect reducing available void spaces. Similarly, the pattern continues in P10F9, P20F9, and P30F9, where elevated FA content correlates with reduced water absorption due to the filling effect and overall porosity reduction. Comparative analysis with neat epoxy, neat epoxy + F6, and neat epoxy + P20 reveals that the addition of PALF generally increases water absorption compared to neat epoxy, possibly due to the hydrophilic nature of these fibres. In general, specimens with higher pineapple fibre content exhibit heightened water absorption, potentially owing to the introduction of additional porosity or hydrophilic characteristics. Conversely, the incorporation of FA tends to diminish water absorption, indicating its potential role in reducing porosity and enhancing water resistance. Further, the water absorption also decreased over a period of five days owing to saturation of the affinity of the fibres to absorb water, and also the micro-coring and closure of the void spaces and micro-gaps between the fibres and the matrix brought about by the FA filler. In summary, the water absorption trends reflect the intricate interplay between pineapple fibre and FA, where pineapple fibre tends to enhance water absorption, and FA exhibits a filling effect, leading to decreased water absorption, with specific outcomes contingent on the wt% of these additives in the epoxy matrix. Fig. 15 gives a graphical representation of the % water absorption for different samples with error bars over a duration of 5 days for every 24 h interval. The results of the present work are interpreted using the findings of M. K. Kumar *et al.* The water absorption test involved immersing the samples in distilled water at room temperature, with specimen weights measured and recorded every 24 hours. Following 432 hours of water immersion, saturation in water absorption and thickness swelling were noted in all composite samples. In comparison to neat epoxy, the inclusion of reinforcements led to an increase in moisture absorption. The composite with optimum wt% of natural reinforcement and filler component demonstrated a noteworthy reduction in water absorption, which was ascribed to consistent mixing and enhanced bonding between the reinforcements and the matrix.^58^

### 3.8 Scanning electron microscopy (SEM)

The morphological investigation of the P20F6 composite specimen was performed utilizing a TESCAN VEGA 3 machine, with an examining voltage of 10 kV. The SEM images yielded a complete portrayal of the microstructure of the composite material, explaining the dispersion and direction of PALF and FA inside the epoxy framework.

**Fig. 16:**
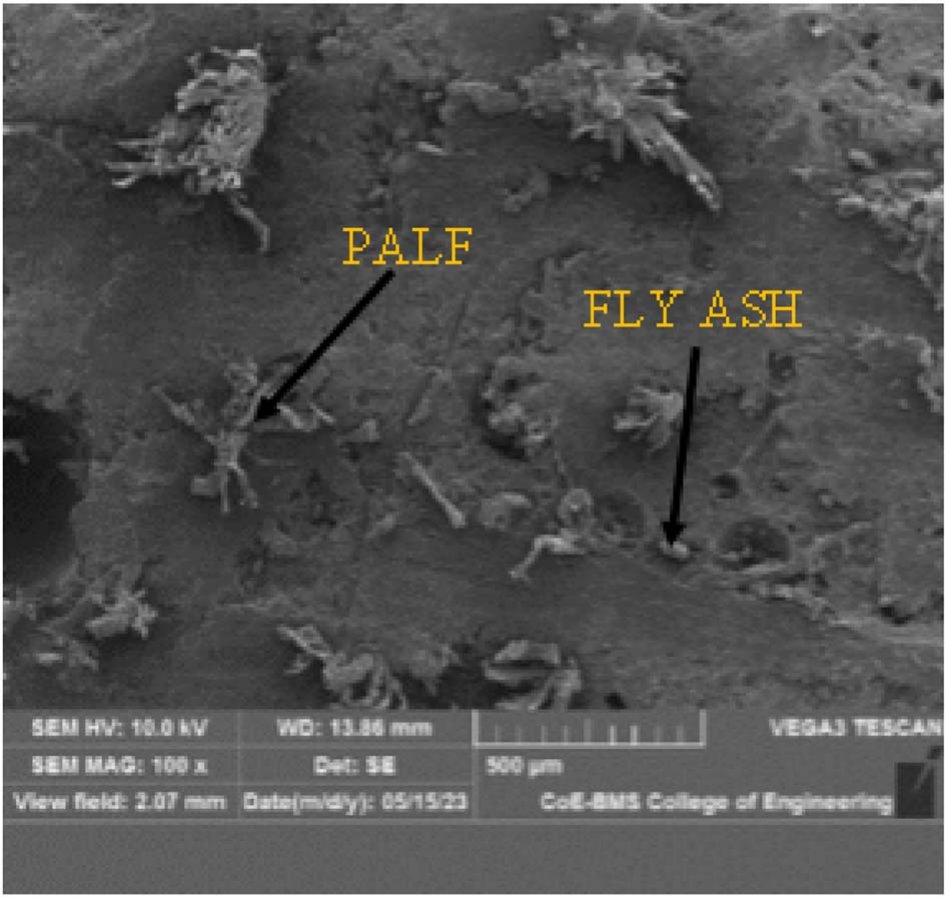
SEM image of P20F6 composite specimen at 100× magnification.

**Fig. 17:**
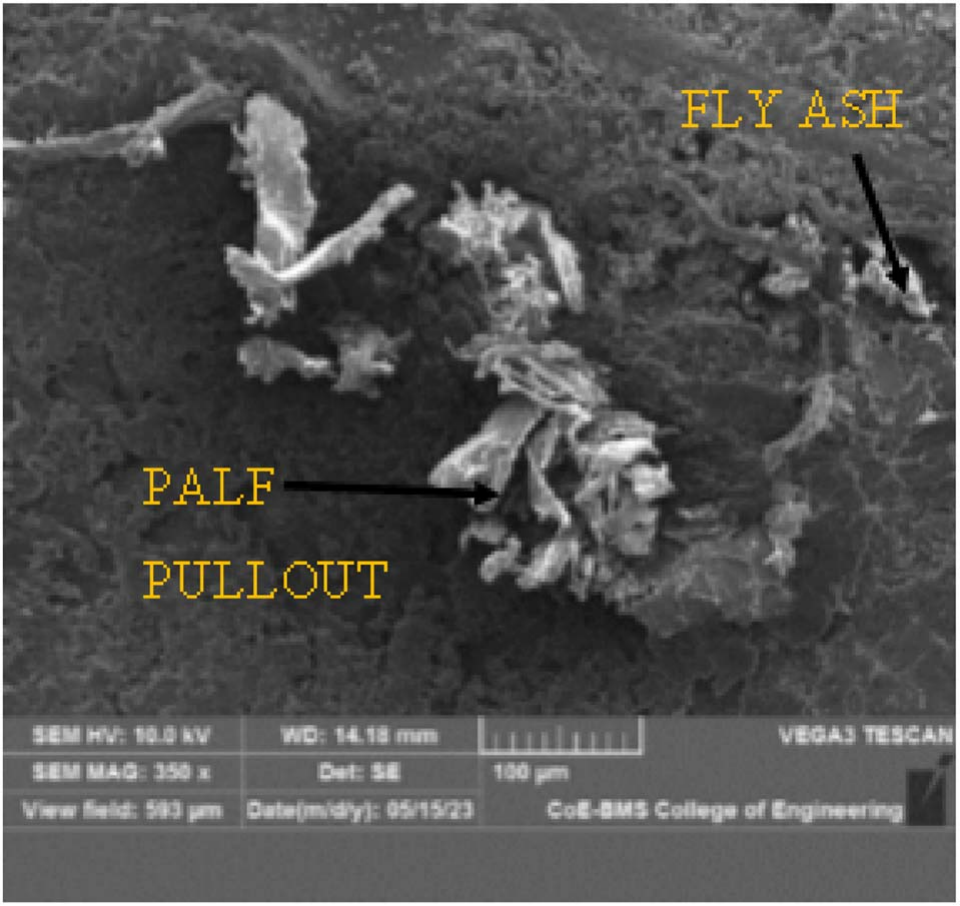
SEM image of P20F6 composite specimen at 350× magnification.

**Fig. 18:**
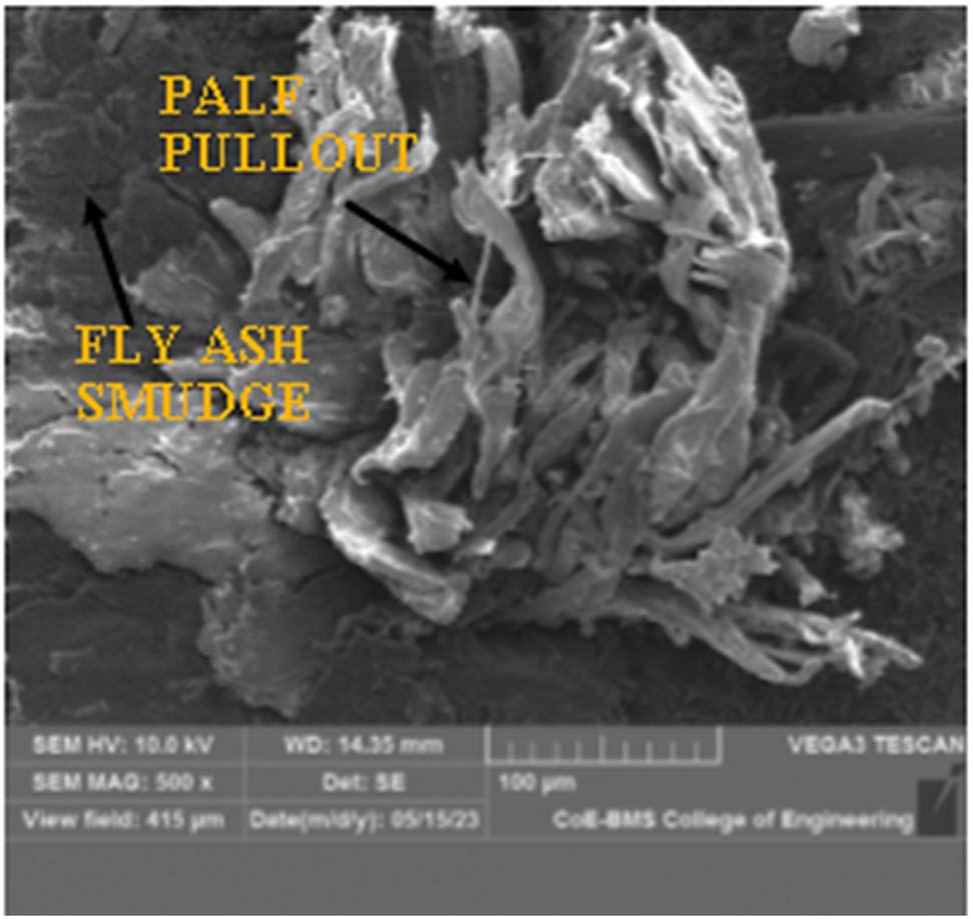
SEM image of P20F6 composite specimen at 500× magnification.

The SEM images give substantial information on the impact of FA on fortification between the framework and the reinforcements. Moreover, it is observed that fracture proliferation and cracking were attributed to the filaments from the lattice owing to laminar debonding, as well as isolation of the grid by pulling out strands. Fig. 16, 17 and 18 elucidate the morphology of the composite with magnifications of 100×, 350×, and 500×, respectively. These pictures successfully portray two critical components: the fibre pullout and the homogeneous dissemination of FA in the matrix network.

## 4. Conclusions

The study demonstrated notable enhancements in PALF-reinforced epoxy composites by the inclusion of FA filler for biomedical applications.

• The tensile strength enhanced with the incorporation of 6 wt% FA surpassed that of neat epoxy by 65.3%. Nevertheless, the tensile strength exhibited a progressive decline beyond this threshold.

• Flexural strength was significantly improved by including FA, resulting in a peak enhancement of 31.9% at 6 wt%. However, a further increase in FA content led to a minor reduction in flexural strength.

• Significant enhancements in impact strength were seen for the addition of 20 wt% PALF and 9 wt% FA, resulting in a stunning 74.18% increase compared to the original epoxy.

• An optimality test conducted with 20.837 wt% PALF and 6.46374 wt% FA predicted results that closely matched the experimental data.

• The Taguchi results revealed that level 2, *viz.*, 6 wt% of FA ranked 1 (higher delta value) and 20 wt% of PALF, yield the maximum UTS from the response tables for SN ratios and means based on the “larger is better” condition.

• Optimality is further validated based on the prediction equations obtained from RSM model evolved using Design-Expert. The % error between the experimental outcomes and predictions for confirmation experiments for UTS is less than 2%, which confirms the RSM model and justifies the results of the optimality test.

• The investigation demonstrated a decline in water absorption when FA was added, indicating enhanced water resistance in the composites. The water absorption of the P10F6 composite was found to be at a minimum of 1.1% on the fifth day, which is nearly equivalent to the water absorption rate of pure epoxy.

• Morphological analysis revealed that FA was uniformly dispersed within the epoxy matrix, indicating strong interfacial bonding between PALF and epoxy. This uniform dispersion significantly contributed to the enhancement of mechanical properties, improved load transfer, and increased resistance to crack propagation.

In summary, the study highlights the use of FA as an environmentally friendly filler, which improves the mechanical properties and decreases water absorption in epoxy composites reinforced with PALF. This environmentally conscious technique promotes the use of agricultural waste for economically feasible and sustainable composite materials that are suited for biomedical applications. It provides insights into the durability and stability of these materials in biological environments.

## Data availability

The necessary data used in the manuscript are already present in the manuscript.

## Author contribution

All authors listed have significantly contributed to the development and the writing of this article.

## Conflicts of interest

The authors declare no conflict of interest.

## Supplementary Material
